# Spontaneous round ligament hematoma as an unusual cause of pelvic pain in a young female patient: MRI demonstration^☆^

**DOI:** 10.1016/j.radcr.2022.02.079

**Published:** 2022-03-25

**Authors:** Pitrone Pietro, Marino Maria Adele, Di Fabrizio Donatella, Cattafi Antonino, Antonuccio Pietro, Sturlese Emanuele, Blandino Alfredo, Ascenti Giorgio, Sofia Carmelo

**Affiliations:** a Section of Radiological Sciences, Department of Biomedical Sciences and Morphological and Functional Imaging, University of Messina, Policlinico "G. Martino" Via Consolare Valeria 1, Messina 98100, Italy; b Department of Human Pathology of Adult and Childhood "Gaetano Barresi", Unit of Pediatric Surgery, University of Messina, Policlinico "G. Martino" Via Consolare Valeria 1, Messina 98100, Italy; c Department of Obstetrics and Gynecology, University of Messina, Policlinico "G. Martino" Via Consolare Valeria 1, Messina 98100, Italy

**Keywords:** Pediatric pelvic pain, Spontaneous round ligament hematoma, MRI

## Abstract

The cause of pelvic pain remains a significant diagnostic challenge, even for experienced radiologists. An accurate differential diagnosis has to be done according to the patient's age and gender. Spontaneous round ligament hematoma is an uncommon cause of acute pelvic pain in adult female patients. To the best of our knowledge, it has never been reported in the literature in the paediatric population. Ultrasound examination is the first line imaging modality for pelvic pain evaluation in young women but it might result inconclusive. Thanks to its panoramic view and multiparametric approach, the MRI can play a pivotal role in the diagnosis of spontaneous round ligament hematoma in paediatric female patients, resulting in a more effective patient's therapeutic management.

## Introduction

Pelvic pain in paediatric population is one of the most common causes of admission to the hospital through the Emergency Department (ED) or Urgent Care (UC). It still represents a diagnostic challenge, even for experienced radiologists.

An accurate differential diagnosis needs to be carried out according to the patient's age and gender. The differential diagnosis for pelvic pain in an adolescent female is broad, ranging from gastrointestinal (eg, appendicitis, chronic inflammatory intestinal diseases, colitis, etc) to genito-urinary (eg, cystitis, reno-ureteral lithiasis, ovarian torsion, endometriosis, etc) conditions [1].

Clinical examination, laboratory tests and imaging examinations are crucial to reach a proper diagnosis. A detailed reproductive history, specifically in young women patients presenting with pelvic pain, should also be taken into consideration [1], [2].

Spontaneous round ligament hematoma is an extremely rare cause of acute pelvic pain in adult female patients [3], [4], [5] and, to the best of our knowledge, such a case has never been reported in the medical literature for the paediatric population.

Imaging plays a pivotal role in the diagnosis and the therapeutic management of this condition in paediatric female patients presenting acute pelvic pain.

## Case report

A 15-year-old girl was admitted at a different hospital reporting recurrent episodes of severe intermittent pain at the right iliac fossa that began three days prior.

The young patient reported normal, painless, menstrual cycles, and her blood tests were unremarkable. No recent trauma nor sexual activity were reported.

The patient performed a lower abdomen ultrasound which showed a large inhomogeneous hypo-anechoic mass (estimated diameter = 10 cm) in the right iliac fossa. Therefore, the patient was referred to our institution where she was scheduled for a contrast-enhanced MRI of the pelvis.

**Fig. 1:**
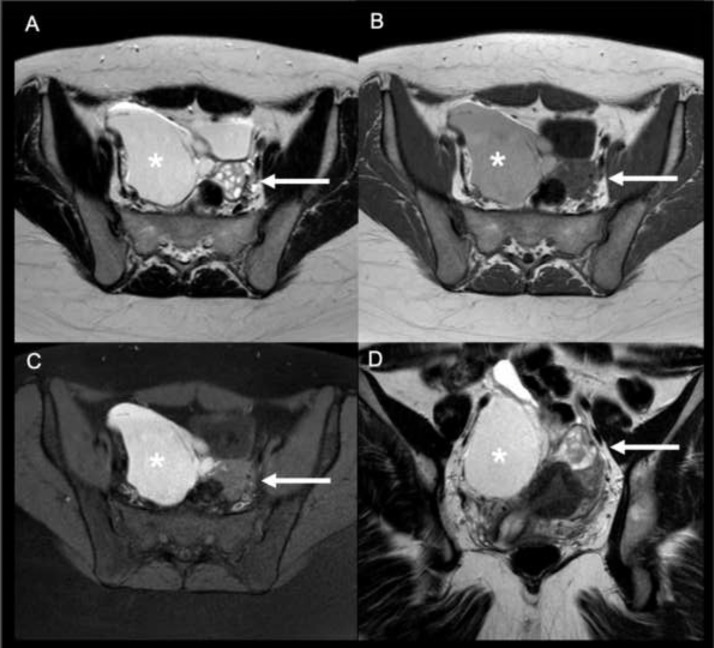
Axial T2-weighted TSE (A), axial T1-weighted TSE (B), axial T1-weighted SPIR (C) and coronal T2- weighted TSE (D) scans of the pelvis. The lesion (*) appears partially hyperintense on T1 and T2 weighted scans due to the proteinaceous-haemorrhagic content. Note the right ovary shifted to the left side (arrows).

**Fig. 2:**
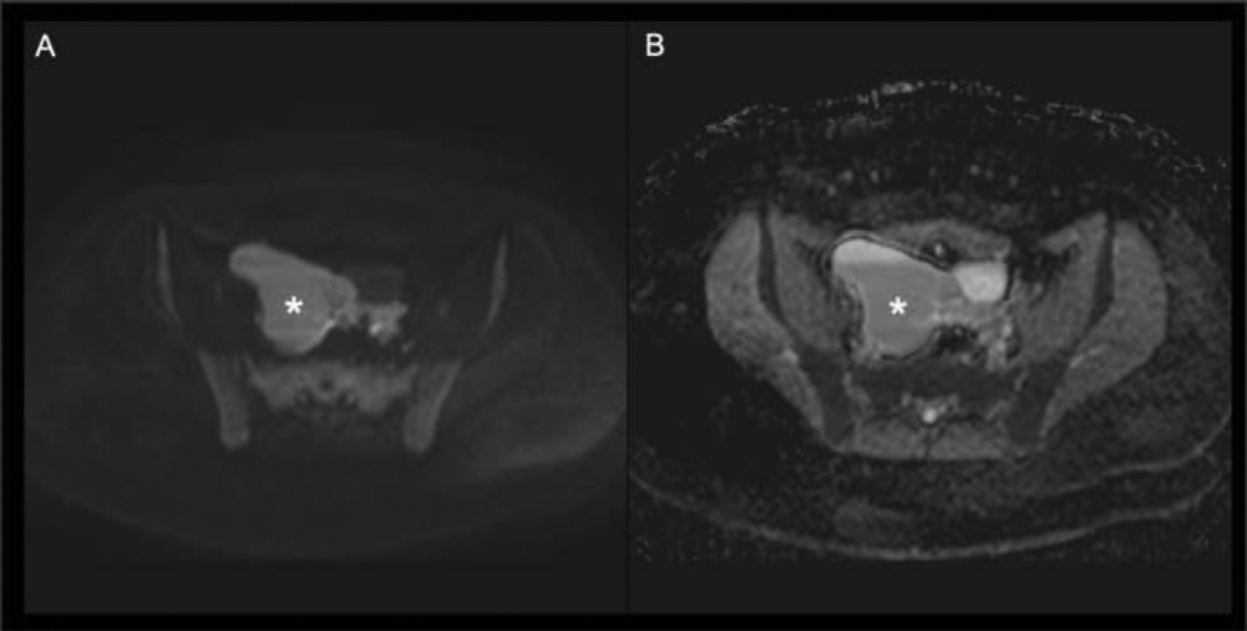
Axial high-b value (1000 sec/mm^2^) DWI- weighted image (A) and corresponding ADC map (B). Note diffusion restriction of the lesion (*) on DWI-weighted image due to the high viscosity of its content. On the ADC map a fluid-fluid level is clearly seen.

On un-enhanced sequences, the right side of the pelvic cavity was occupied by a large (estimated diameter = 10 cm) fluid collection, which was appearing hyperintense on T1w, T2w and high-b value (1000 sec/mm^2^) DWI- weighted images (Fig. 1, Fig. 2). Multiple septations were seen on the fluid collection's superior-medial aspect. These findings confirmed the proteinaceous-haemorrhagic content of the collection.

**Fig. 3:**
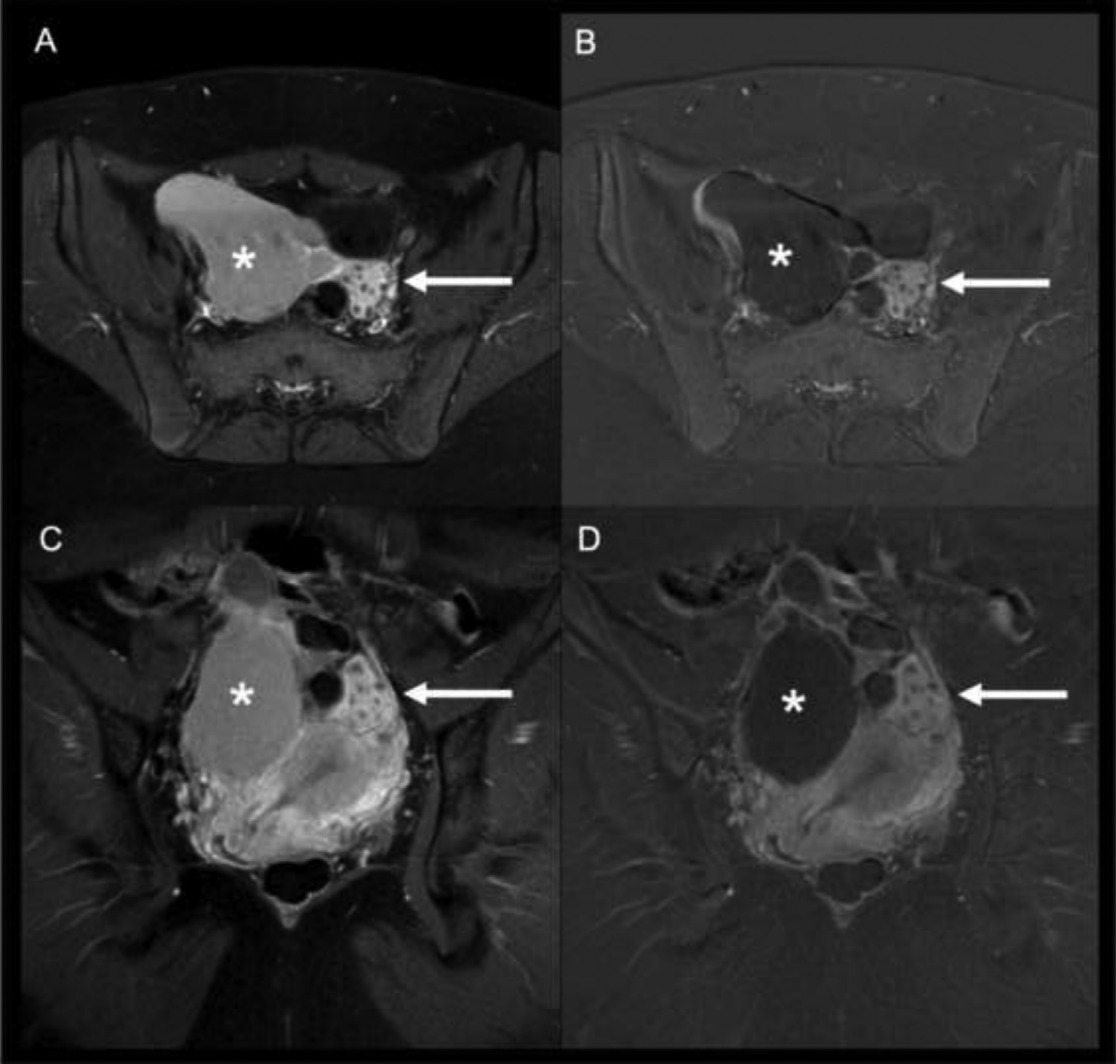
T1 TSE SPIR axial (a) and coronal (c) post-contrast images and corresponding subtracted images. Minimum contrast enhancement of the septa with no macroscopic nodulations are seen within the lesion (*). The right ovary is contralaterally shifted (arrows).

The right ovary appeared contralaterally shifted, and a small amount of free fluid was identifiable in the pelvic gutter. Post-contrast scans (Fig. 3) showed a minimum contrast enhancement of the septa with no macroscopic nodulations within the lesion.

In order to manage the patient's symptoms and in absence of a clear diagnosis, the paediatric surgeon decided to perform a diagnostic video-laparoscopy: a hematoma within the right round ligament was detected and promptly drained.

Immediate relief of symptoms was observed, with the patient being dismissed a few days later. Neither short nor long-term surgical complications were reported.

## Discussion

Round ligament represents one of the visceral layers of the pelvic fascia (fused with parametrium) and provides, along with other ligaments and muscles of the pelvic floor, stability to this region and support of the pelvic viscera. Medical literature on the round ligament's bleeding remains poor [3], [4], [5], and no paediatrics cases have been yet described.

In 1982, Borrero reported the case of a 43-year-old woman with a groin painful mass appearing on straining mimicking an incarcerated inguinal hernia [3]. After ruling out a pregnancy, the patient underwent exploratory surgery that revealed an unexplained haemorrhage into the round ligament.

More recently, Kim and colleagues in a retrospective study of 59 patients diagnosed with postpartum hemorrhage found that non-uterine arteries, including the arteries running within the round ligament, are the major sources of post-partum haemorrhage and, thus, an important target for embolization [4].

Some authors also reported cases of hemoperitoneum following the bleeding of Sampson's artery in patients with history of inguinal hernia repair [5]. Sampson's artery, that usually obliterates in postembryonic development, courses along the round ligament of the uterus in the inguinal canal of females, originating from the arcade between the uterine and ovarian arteries, and can lead to hemoperitoneum in case of disruption. Woman of childbearing age, particularly those in the peripartum period, are more likely to have this rare complication [5].

Mine et al [6]. reported 10 cases of pregnant women suspected to suffer from inguinal hernias at a clinical examination. After colour-Doppler US, round ligament varicosities with no clear signs of bleeding were diagnosed. All patients were treated with conservative management, and the symptoms resolved, in all cases, after delivery.

Round ligament can be also involved by endometriosis: in those cases, the round ligament appears thickened or nodular, with or without small internal T1w hyperintense haemorrhagic foci [7].

As far as we know, no spontaneous round ligament haematoma has been yet reported in paediatric female patients.

A specific diagnosis of round ligament hematoma based on ultrasound examination is not possible: US can demonstrate an inhomogeneous cystic lesion containing debris occupying part of the pelvic cavity. Vermiform appendix and normal ovaries should be always identified when an US is performed in a female patient.

Thanks to its panoramic view and multiparametric approach, the MRI facilitates a better definition of the anatomical structures of the pelvis as well as the tissue characterization, which is based on signal intensities on different sequences. Furthermore, MRI has no radiation exposure. Hence, it represents the appropriate method for a correct diagnosis. On MRI, a recent round ligament hematoma appears hyperintense on T2w and on T1w images with and without fat suppression due to the paramagnetic effect of methaemoglobin. On high-b value (1000 sec/mm^2^) DWI- weighted images, the hematoma shows a diffusion restriction due to the high viscosity related to the proteinaceous and haemoglobin degradation products’ content [8]. On the ADC map (Fig. 2 B) a fluid-fluid level related to the different densities of blood products is well demonstrated: the heavier cellular components show a gravity dependent position whereas the plasma fluid lies on the upper aspect of the haematoma (the so called “haematocrit effect”) [9].

In a female patient, the presence of a large haemorrhagic cystic lesion localized in the pelvis (within the presumptive site of the round ligament), which dislocates the ovary otherwise normal in morphology and signal, should raise the suspicion of round ligament haematoma in case of no signs of deep pelvic endometriosis.

Hence, surgical exploration and drainage might be needed.

## Conclusion

When dealing with female adolescents suffering from acute pelvic pain, in the absence of any other potential causes, spontaneous round ligament hematoma should be considered as a rare but potential diagnosis.

US is the first line imaging modality in young female patients having pelvic pain, but it might result inconclusive. In such cases, MRI is a problem-solving technique that can guide an effective patient's treatment management.

## Patient consent

The patient provided a written informed consent for using anonymized data for publication.

## Declarations

All procedures performed in the study were in accordance with the ethical standards of the institutional and/or national research committee and with the 1964 Helsinki declaration and its later amendments or comparable ethical standards. Consent to participate: not applicable for this type of the work (case report).
