# Corrigendum to “Spontaneous round ligament hematoma as an unusual cause of pelvic pain in a young female patient: MRI demonstration” [Radiology Case Reports 17 (2022) 1765–1769]

**DOI:** 10.1016/j.radcr.2022.06.023

**Published:** 2022-07-08

**Authors:** Pietro Pitrone, Maria Adele Marino, Donatella Di Fabrizio, Antonino Cattafi, Pietro Antonuccio, Emanuele Sturlese, Alfredo Blandino, Giorgio Ascenti, Carmelo Sofia

**Affiliations:** a Section of Radiological Sciences, Department of Biomedical Sciences and Morphological and Functional Imaging, University of Messina, Policlinico ``G. Martino'' Via Consolare Valeria 1, Messina 98100, Italy; b Unit of Pediatric Surgery, Department of Human Pathology of Adult and Childhood ``Gaetano Barresi'', University of Messina, Policlinico ``G. Martino'' Via Consolare Valeria 1, Messina 98100, Italy; c Department of Obstetrics and Gynecology, University of Messina, Policlinico "G. Martino" Via Consolare Valeria 1, Messina 98100, Italy

The authors would like to inform you that their names were inverted in their published article with last names appearing before first names. This has been corrected above.

